# Identification of drought-response genes and a study of their expression during sucrose accumulation and water deficit in sugarcane culms

**DOI:** 10.1186/1471-2229-11-12

**Published:** 2011-01-13

**Authors:** Hayati M Iskandar, Rosanne E Casu, Andrew T Fletcher, Susanne Schmidt, Jingsheng Xu, Donald J Maclean, John M Manners, Graham D Bonnett

**Affiliations:** 1 CSIRO, Plant Industry, Queensland Bioscience Precinct, 306 Carmody Road, St. Lucia, QLD, 4067, Australia; 2 School of Chemistry and Molecular Biosciences, University of Queensland, St. Lucia, QLD, 4072, Australia; 3 School of Biological Sciences, University of Queensland, St Lucia, QLD, 4072, Australia; 4 Indonesian Biotechnology Research Institute for Estate Crops, Jl. Taman Kencana No.1, Bogor 16151, Indonesia; 5 The Key Laboratory of Eco-physiology and Genetic Improvement for Sugarcane, Ministry of Agriculture, Sugarcane Institute of Fujian Agriculture and Forestry University, Fuzhou, 350002, Peoples Republic of China

## Abstract

**Background:**

The ability of sugarcane to accumulate high concentrations of sucrose in its culm requires adaptation to maintain cellular function under the high solute load. We have investigated the expression of 51 genes implicated in abiotic stress to determine their expression in the context of sucrose accumulation by studying mature and immature culm internodes of a high sucrose accumulating sugarcane cultivar. Using a sub-set of eight genes, expression was examined in mature internode tissues of sugarcane cultivars as well as ancestral and more widely related species with a range of sucrose contents. Expression of these genes was also analysed in internode tissue from a high sucrose cultivar undergoing water deficit stress to compare effects of sucrose accumulation and water deficit.

**Results:**

A sub-set of stress-related genes that are potentially associated with sucrose accumulation in sugarcane culms was identified through correlation analysis, and these included genes encoding enzymes involved in amino acid metabolism, a sugar transporter and a transcription factor. Subsequent analysis of the expression of these stress-response genes in sugarcane plants that were under water deficit stress revealed a different transcriptional profile to that which correlated with sucrose accumulation. For example, genes with homology to late embryogenesis abundant-related proteins and dehydrin were strongly induced under water deficit but this did not correlate with sucrose content. The expression of genes encoding proline biosynthesis was associated with both sucrose accumulation and water deficit, but amino acid analysis indicated that proline was negatively correlated with sucrose concentration, and whilst total amino acid concentrations increased about seven-fold under water deficit, the relatively low concentration of proline suggested that it had no osmoprotectant role in sugarcane culms.

**Conclusions:**

The results show that while there was a change in stress-related gene expression associated with sucrose accumulation, different mechanisms are responding to the stress induced by water deficit, because different genes had altered expression under water deficit.

## Background

Sugarcane (*Saccharum *spp.) is a C_4 _grass with a characteristic ability to accumulate high sucrose concentrations in the culm. Sucrose is synthesized in the leaf mesophyll and transported via the phloem primarily through symplastic transport into storage parenchyma [1]. Accumulation of sucrose in the culm is the net result of sucrose import from the leaf, metabolism within the culm and sucrose export from culm tissue [2]. Sugarcane culm tissues can accumulate sucrose to a concentration of approximately 650 mM in storage parenchyma [3]. It has been suggested that the accumulation of sucrose in the storage parenchyma to such a high concentration may cause metabolic stress to tissues and cellular compartments in sugarcane culms. It may also create steep osmotic gradients between compartments with varying sucrose concentrations [4]. Therefore, cells in the culm must adapt to a range of potentials while maintaining metabolism [4].

Previously, numerous genes with various functions were identified as being differentially expressed between immature culm tissue with low sucrose content and mature culm tissue with high sucrose content through analyses of expressed sequence tags (ESTs) [5] and microarray-derived expression data [6,7]. Transcripts associated with protein synthesis and primary metabolism were more abundant in immature culms, while transcripts corresponding to genes associated with fibre biosynthesis and abiotic stress tolerance, particularly osmotic and oxidative stress, were more abundant in maturing culms [7]. However, genes encoding proteins with known functions related to sucrose metabolism were not highly expressed in culm tissues irrespective of sucrose content [6]. Casu et al. [8] proposed that sucrose accumulation may be regulated by a network of genes induced during culm maturation which included clusters of genes with roles that contribute to key physiological processes including sugar translocation and transport, fibre synthesis, membrane transport, vacuole development and function, and abiotic stress tolerance. Recently, Papini-Terzi et al. [9] compared the results of a microarray-based expression analysis of 30 sugarcane genotypes with variation in sugar content (measured as Brix) with that of an earlier study [10] of signal transduction-related gene expression under water deficit and treatment with the stress-related hormone abscisic acid (ABA). There was considerable overlap between signalling genes associated with sugar accumulation and those involved in drought adaptation but less so with ABA treatment [9]. Thus, a more detailed comparison of the expression of stress-responsive genes in relation to sucrose accumulation and water deficit is warranted.

To maintain turgor or pressure potential under osmotic stress, plants synthesise metabolites such as sugars, polyols, amino acids and betaines that have a role in protecting membranes and maintaining osmotic potential [11,12]. As compatible solutes or osmoprotectants, these metabolites may have a role in adaptation to protect metabolism under conditions of high solute concentration such as that present in sugarcane storage cells. If the sucrose content in the cytoplasm of storage parenchyma is low, some stress-related genes including those involved in the synthesis of osmoprotectants, may have a role in protecting the cells, and maintain pressure potential by providing compatible solutes in the cytoplasmic compartment. Alternatively, if the sucrose content in the cytoplasm is high, osmoprotectants as well as protein chaperones may be involved in the protection of protein and membrane structure in the cytoplasm. At the molecular level, a number of genes in plants that are induced by osmotic stress, some with roles in osmoprotection, have been identified and characterized, and the function of these genes have been examined through the use of transgenic plants of various species to demonstrate their role in stress tolerance [13,14].

The expression of stress-related genes in different parts of the sugarcane culm raises the question of the role of these genes in maturing sugarcane internodes. One hypothesis is that the expression of stress-related genes, and the consequential cellular responses, would facilitate the accumulation of high levels of sucrose. This study investigated whether the degree of expression of stress-related genes, was correlated with the sucrose content in the sugarcane culm, and whether such genes were also responsive to water deficit stress. Therefore, known stress-related genes were selected for expression analysis to identify genes with transcript levels that correlated with sugar content in culm and leaf tissues. Expression patterns of a sub-set of these genes that were associated with sucrose accumulation were analysed across 13 genotypes of sugarcane and its relatives to further test the correlation of gene expression with sucrose accumulation in the culm. The expression of this sub-set of genes was subsequently examined in plants of one cultivar undergoing water deficit. The functional identity of these genes provides a basis for the prediction and comparison of mechanisms that potentially allow the accumulation of sucrose to high levels in sugarcane and tolerance to water deficit.

## Results

### Stress-related gene expression and sucrose content at different developmental stages

#### Analysis of sugars

Stem and leaf tissues derived from mature plants of the cultivar Q117 were analysed for their content of three relevant sugars. Glucose and fructose concentrations were both lower in the last fully-expanded leaf (LFE) and more mature internodes (I13-14) than I4-5 and I7-8 (Table 1). The concentration of sucrose was lowest in the leaf and I4-5, and increased down the culm to a concentration of 125 mg per g FW in internodes 13-14. These changes in sucrose concentration were in accordance to previous analyses of changes of sucrose during sugarcane growth and development [15].

**Table 1:** Concentration of sucrose, glucose and fructose on a fresh weight basis in sugarcane tissues of cultivar Q117 as measured by HPLC.

**Tissue**^**1**^	Sugars (mg/g FW) ± SE		
	
	Sucrose	Glucose	Fructose
LFE	13.17 ± 2.48^a^	1.72 ± 0.42^a^	2.14 ± 0.73^a^
I4-5	9.07 ± 3.42^a^	14.59 ± 2.07^b^	12.68 ± 1.86^b^
I7-8	44.87 ± 6.44^b^	14.87 ± 1.03^b^	11.47 ± 1.01^b^
I13-14	124.84 ± 12.97^c^	1.07 ± 0.36^a^	1.21 ± 0.36^a^

^1^LFE = last fully expanded leaf, I4-5 = internodes 4 and 5, I7-8 = internodes 7 and 8, I13-14 = internodes 13 and 14. Means are shown ± standard error of the mean (SE) (n = 3).

^a^Within a column, numbers with the same letter are not significantly different based on LSD test from one-way analysis of variance (P ≤ 0.05).

#### Expression of stress-related genes

The relative abundance of transcripts of the 51 genes in different parts of mature plants of cultivar Q117 was determined using Real Time quantitative PCR (RT-qPCR) analysis of total RNA samples derived from the LFE, I4-5, I7-8 and I13-14. Transcript expression levels were standardized to transcripts of *GAPDH *as a reference gene because this gene is known to be expressed at a relatively constant level in leaves, and immature and mature internodes of cultivar Q117 [16].

Differential expression was observed between the leaf and different culm tissues for transcripts encoding proteins with probable roles in amino acid, polyamine, sugar and polyol metabolism, sugar transport, chaperone functions and transcriptional regulation. Of the 51 genes tested, 17 genes showed higher expression in the most mature internodes of the culm than in the leaf. Of these 17 genes, nine were more highly expressed in the older internodes, I13-14, compared to the younger internodes, I4-5 (Table 2).

**Table 2:** RT-qPCR expression analysis of 32 stress-related genes showing significant differential expression in tissues from sugarcane variety Q117.

Gene	Mean				
	**P value**	**LFE**^**1**^	**I **_**4-5**_	**I **_**7-8**_	**I **_**13-14**_

*Up-regulated Trehalase*	<.001	0.0102^a^	0.0230^b^	0.0240^b^	0.0370^c^
*TAP24F-4*	<.001	0.1241^a^	1.1617^c^	0.7071^b^	0.6160^b^
*TM89-33*	0.002	0.0659^a^	0.1502^c^	0.1059^b^	0.1418^c^
***TF1***^***2***^	**0.009**	**0.0395**^**a**^	**0.1370**^**b**^	**0.1238**^**b**^	**0.1866**^**b**^
*TM11b*	0.0342	0.0105^a^	0.0217^b^	0.0195^ab^	0.0250^b^
***Pox***	**<.001**	**0.1511**^**ab**^	**0.1127**^**a**^	**0.1879**^**b**^	**0.2798**^**c**^
***OAT***	**<.001**	**0.0205**^**a**^	**0.0199**^**a**^	**0.0201**^**a**^	**0.0872**^**b**^
***AS***	**<.001**	**0.0126**^**a**^	**0.1024**^**b**^	**0.1159**^**b**^	**0.1591**^**c**^
*Samsynt*	<.001	1.9011^a^	11.9409^b^	10.8635^b^	13.0849^b^
*SPDS*	0.0108	0.0785^a^	0.0744^a^	0.0935^ab^	0.1056^b^
*PST2a*	<.001	0.0566^a^	3.2513^b^	3.4418^b^	2.5815^b^
*PST2b*	0.008	0.0369^a^	0.1816^b^	0.2064^b^	0.1904^b^
***PST5***	**0.021**	**0.0141**^**a**^	**0.0688**^**ab**^	**0.1428**^**bc**^	**0.2272**^**c**^
*PST7*	<.001	0.0577^a^	0.2210^c^	0.1330^b^	0.1302^b^
***LEA***	**<.001**	**0.0415**^**a**^	**0.0755**^**a**^	**0.0826**^**a**^	**0.3197**^**b**^
***Dehydrin***	**0.0442**	**0.0074**^**a**^	**0.0158**^**a**^	**0.0088**^**a**^	**0.0454**^**b**^
*ABC transporter*	0.025	0.0252^a^	0.0490^ab^	0.0408^a^	0.0665^b^
***P5CS***	**0.056**	**0.1093**^**a**^	**0.1424**^**a**^	**0.1465**^**a**^	**0.2511**^**a**^
*Down-regulated Gols*	0.009	0.1009^b^	0.0571^a^	0.0172^a^	0.0092^a^
*TPP*	0.003	0.8318^b^	0.4294^a^	0.3904^a^	0.3016^a^
*DREB-like protein*	0.02	0.0016^a^	0.0057^b^	0.0022^a^	0.0019^a^
*THB43-11*	<.001	0.3665^b^	0.1105^a^	0.1361^a^	0.1502^a^
*HvDRF1*	<.001	0.4607^c^	0.2112^b^	0.1223^a^	0.1409^a^
*TWC1*	<.001	0.0529^b^	0.0280^a^	0.0200^a^	0.0206^a^
*ShSUT1*	0.001	0.1434^b^	0.1261^b^	0.0697^a^	0.0481^a^
*DnaJ*	<.001	0.2349^a^	0.9842^c^	0.6780^b^	0.4610^ab^
*HPPase*	<.001	0.0105^a^	0.3023^b^	0.0350^a^	0.0270^a^
*Osmotin*	0.003	0.0503^a^	0.2302^b^	0.0572^a^	0.0577^a^
*Stress-related protein*	0.002	0.0997^a^	0.3784^b^	0.1460^a^	0.1152^a^
*Expansin*	<.001	0.3810^a^	4.1719^b^	0.1148^a^	0.0389^a^
*Lipoxigenase*	<.001	0.0013^b^	0.0002^a^	0.0002^a^	0.0003^a^
*PEAMT*	0.016	0.2207^b^	0.2079^b^	0.0979^a^	0.0926^a^
*ADI*	<.001	0.0153^a^	0.0251^c^	0.0194^b^	0.0181^ab^

^1^The tissues are as described in Table 1. The expression of each gene was normalised relative to that of *GAPDH*.

^2^The eight genes selected for analysis in further experiments are shown in bold typeface. (See additional file 2 for full name of each gene and primers sequences). Data for *PC5S *(P = 0.056) which was included in further experiments (see text) is also presented.

^a^Mean values within a gene with the same letter are not significantly different based on LSD test (P ≥ 0.05) from one-way analysis of variance (ANOVA).

Genes with statistically significant up-regulated expression in older internodes when compared to young internodes (I4-5) were those encoding the putative chaperones dehydrin and late embryogenic abundant (LEA) protein; enzymes involved in proline metabolism, ornithine aminotransferase (OAT) and proline oxidase (Pox); the trehalose degradative enzyme, trehalase; a spermidine synthase gene (SPDS); and asparagine synthase (AS). Transcription factors with bZIP (TF1), myb (TM89-33; TM11b) or ERF (TAP24F-4) family domains were also more highly expressed in the older culm internodes than the leaf. Genes encoding the sugar transporters PST5, PST7, PST2a and PST2b were more highly expressed in the mature internodes than leaf. Expression level of *PST5 *increased down the culm, while, *PST7 *was expressed at a higher level in I4-5 than in I13-14. Fifteen of the genes were down-regulated in at least one part of the culm when compared to the leaf. For example, in contrast to the other sugar transporters, the sucrose transporter ShSUT1 was up-regulated in the leaf and the young internodes (I4-5) when compared to the mature culm. The remaining genes did not show altered expression levels in the leaf compared to the culm. Of these, the tonoplastic intrinsic protein (TIP) was previously shown to be down-regulated in more mature internodes [10] but not in this study.

Eight genes were selected to further examine their relationship with sucrose content. *OAT*, *Pox*, *AS*, *LEA*, *dehydrin*, and *PST5 *genes were selected as they were all up-regulated in the older culm internodes. Since the *OAT *and *Pox *genes are both involved in proline metabolism, *P5CS *was also included in the selected genes as it catalyzes the synthesis of a primary precursor for proline biosynthesis in plants and also showed a trend (P = 0.056) of increased expression with culm maturity. The bZIP transcription factor-encoding gene *TF1 *was also included in this group as it was the only gene encoding a transcription factor that showed a trend to increased expression in the most mature internode, and also had relatively low expression in the leaf compared to the culm.

### Expression of the eight selected genes and amino acid content in sugarcane genotypes varying in sugar accumulation

#### Gene expression

**Figure 1:**
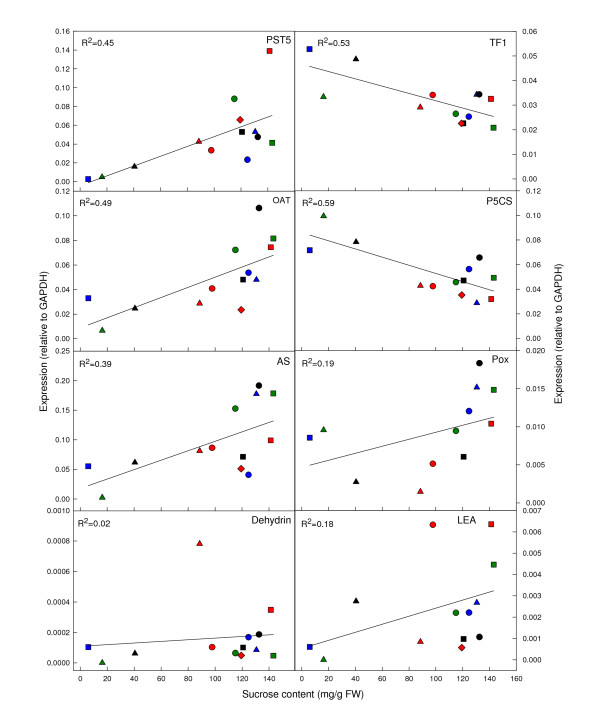
**Correlation of gene expression with sucrose content**. Relative expression of *PST5*, *OAT, AS*, *dehydrin*, *TF1*, *P5CS*, *Pox *and *LEA *plotted against sucrose content of the lowest internodes (I13-14) of 13 different genotypes. Gene expression is normalised to transcripts of *GAPDH *and the average value (n = 3) was plotted for each genotype, Q28 (red circle), Q117 (green square), Q124 (red square), Q165 (green circle), Q200 (black circle), Badilla (red triangle), IJ76-237 (red diamond), IJ76-567 (blue circle), NG51-99 (black square), NG77-98 (blue triangle), Mandalay (black triangle), SES 106 (blue square) and *Erianthus *(green triangle). The R^2 ^for each gene is also shown.

Expression of the eight selected genes was determined for several sugarcane varieties and closely-related sugarcane progenitor genotypes varying in sucrose content. The sucrose content of the most mature internodes sampled from plants ranged from 6 - 143 mg/g FW. RNA was also isolated from the lowest culm internodes (I13-15). The expression levels of *OAT *(R = 0.698), *PST5 *(R = 0.670) and *AS *(R = 0.626) transcripts were positively correlated (P ≤ 0.05) with sucrose content, whilst those of *P5CS *(R = -0.768) and *TF1 *(R = -728) were significantly (P ≤ 0.05) negatively correlated with sucrose content (Figure 1). However, *dehydrin*, *LEA *and *Pox *transcript levels had no significant correlation with sucrose content (R = 0.124 - 0.432) (Figure 1).

#### Amino acid content

In the previous experiment, a number of transcripts encoding enzymes involved in amino acid metabolism (OAT, AS, P5CS) showed significant relationships with sucrose content. Therefore, free amino acids were measured to determine any changes in the metabolite pool in relation to sucrose content. The analysis was initially conducted by HPLC because this method has been used previously to measure free amino acids in sugarcane [17]. This analysis also avoided the interference caused by high sucrose content when using biochemical or colorimetric assays [18,19]. Tejera et al. [17] reportedly measured seventeen amino acids using this method (Asp, Ser, Glu, Gly, His, Arg, Thr, Ala, Pro, Tyr, Val, Met, Lys, Ile, Leu and Phe), however, several amino acids were not detected by this HPLC method. Further analysis showed that, based on retention time, proline co-eluted with γ-amino butyric acid (GABA), asparagine with serine, histidine with glutamine, and threonine with citrulline. This was a particular problem as accurate measurement of proline was essential. These analyses suggest that the methodology used by Tejera et al. [17] was not suitable for our purpose and other methods were sought.

Consequently, samples were tested by UPLC which has greater resolving power. Twenty amino acids were measured by UPLC in the most mature internodes from diverse sugarcane genotypes (additional file 1). The five amino acids with the highest concentrations in almost all genotypes were Asn, Gln, Ser, GABA and Glu. Moreover, Q28 had much higher levels of Asn, Gln, Ala and Val than other genotypes. The results also suggested that there was no major difference in the profile of amino acids between the low and high sucrose content genotypes. Interestingly, Pro concentration was negatively correlated with sucrose content (P ≤ 0.01). Among the 20 protein amino acids analysed, only Pro and Leu were significantly correlated with sucrose content and showed a negative correlation (-0.82 and -0.86, respectively, additional file 1). The result for Pro was in accordance with the negative correlation of expression of the gene encoding the proline biosynthetic enzyme P5CS with sucrose content.

### Gene expression, sugar and amino acid content in sugarcane cultivar Q117 under water deficit stress

#### *Physiological responses to *water deficit *stress*

Sugarcane plants (cultivar Q117) were grown in pots for approximately five months as detailed in the methods and then watering ceased on a sub-set of plants in order to assess the effect of water deficit stress on gene expression. Relative water content (RWC), photosynthetic rate and stomatal conductance were measured to monitor the development of stress. By three days after the cessation of watering, the photosynthetic rate and stomatal conductance of the last fully expanded leaf had dropped to almost zero, indicating that the plants were experiencing very severe stress (data not shown). There were no significant changes in photosynthetic rate and stomatal conductance of the control plants between the start and end of the experiment. RWC of leaves from plants subjected to water deficit stress decreased 3 days after the cessation of watering (data not shown). The photosynthetic rate, stomatal conductance and RWC of the stressed plants decreased progressively over two weeks of water deficit stress while that of the control plants was unchanged (Table 3).

**Table 3:** Stomatal conductance, photosynthetic rate and relative water content (RWC) of the last fully expanded leaf from water deficit stressed and control plants.

	Time 0		15 days	
	
	Control	Water deficit	Control	Water deficit
Stomatal conductance (mmol H_2_O m^-2^s^-1^)	240^a^	220^a^	310^a^	20^b^
Photosynthesis (μmol CO_2_m^-2^s^-1^)	29.91^a^	28.44^a^	29.37^a^	0.14^b^
RWC (%)	98.93^a^	95.86^a^	98.07^a^	43.17^b^

^a^Within each row, numbers with different letters are significantly different (LSD test, one-way analysis of variance, P ≤ 0.05).

#### Sugar content of sugarcane under water deficit stress

Glucose and fructose levels, on a dry weight basis, were similar in all tissues except for the lowest internode (Table 4). Despite the moisture content of the lowest internodes from the different treatments being the same, both glucose and fructose were elevated in the internodes from the plants undergoing water deficit. On a dry weight basis, sucrose content in leaves was greatly reduced three days after imposition of stress conditions (data not shown), and remained lower up to 15 days after water deficit commenced (Table 4). The sucrose content in the culm internodes from plants under water deficit were the same as controls (Table 4). The similar moisture and sucrose contents between mature internodes from plants undergoing water deficit and the well-watered controls means that the responses in metabolism and gene expression in the plants undergoing water deficit were not confounded by changes in sucrose concentration.

**Table 4:** Glucose, fructose and sucrose content in different tissues of sugarcane cultivar Q117 15 days after imposition of water deficit.

	**Glucose**^**2**^ (mg/g DW)		Fructose (mg/g DW)		Sucrose (mg/g DW)		Moisture content (%)	
	
Tissue^1^	Water deficit	Control	Water deficit	Control	Water deficit	Control	Water deficit	Control
LFE	4.98 ± 0.42^a^	4.31 ± 1.27^a^	2.52 ± 0.17^a^	2.71 ± 0.69^a^	5.10 ± 0.25^b^	48.81 ± 1.83^a^	37.3 ± 0.12^a^	66.0 ± 0.58b
M-I2	46.83 ± 9.16^a^	28.41 ± 1.10^a^	45.30 ± 3.95^b^	27.96 ± 0.83^a^	95.37 ± 5.48^a^	104.94 ± 4.39^a^	81.1 ± 0.56^a^	88.9 ± 0.85^b^
I4-5	137.47 ± 10.43^a^	117.39 ± 10.60^a^	106.77 ± 9.81^a^	100.57 ± 10.99^a^	82.24 ± 19.03^a^	131.34 ± 36.46^a^	79.0 ± 0.34^a^	88.9 ± 1.11^b^
I7-8	84.71 ± 5.5^a^	73.75 ± 7.34^a^	66.97 ± 7.38^a^	55.11 ± 5.48^a^	259.46 ± 34.20^a^	396.86 ± 53.67^a^	74.8 ± 1.06^a^	81.7 ± 1.58^b^
LI	16.30 ± 0.55^b^	9.69 ± 1.05^a^	16.17 ± 1.18^b^	11.24 ± 1.22^a^	379.10 ± 24.69^a^	369.38 ± 29.56^a^	69.7 ± 3.51^a^	68.7 ± 3.11^a^

^1 ^LFE = last fully expanded leaf, M-I2 = meristem to internode 2, I4-5 = internodes 4 and 5, I7-8 = internodes 7 and 8, LI = lowest internode.

^2^Data are presented as mean values (n = 3). Between treatment and control for each type of sugars, numbers with different letters are significantly different based on Student t-test (P ≤ 0.05)

#### Expression of stress-related genes in response to water deficit

The expression of the eight genes selected from the previous experiment (Table 2) was compared in plants treated with water deficit stress. Expression analysis was carried out on RNA isolated from the young culm internodes (I4-5) and mature culm (the lowest internodes) which have very different sucrose content.

**Figure 2:**
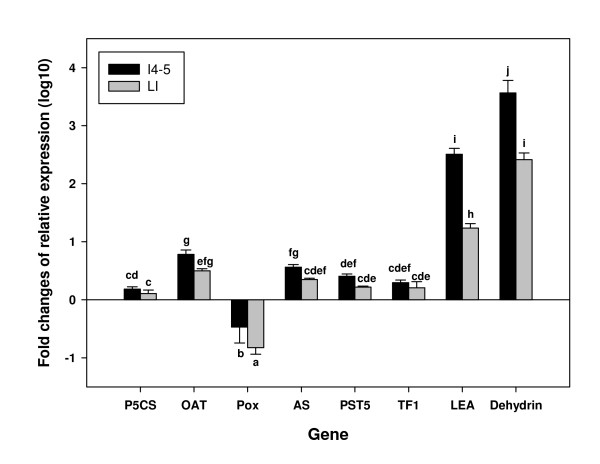
**Response of gene expression to water deficit**. Changes in gene expression of selected stress-related genes under water deficit stress after 15 days of treatment of the internodes 4 and 5 (I4-5) and the lowest internodes (LI). Results are presented as the ratio of expression of each gene (relative to that of *GAPDH*) in water deficit stressed plants compared to controls, transformed in log10. Error bars indicate the standard error of the mean (n = 3). Bars with the same letters are not significantly different based on LSD test from two-way analysis of variance (P ≤ 0.05).

Almost all of the selected genes were up-regulated under the 15-day water deficit stress regime when compared with the well-watered control plants. The exception was *Pox*, which was down-regulated, as may be expected for a catabolic enzyme (Figure 2). Expression of *P5CS, OAT, AS, PST5 *and *TF1 *transcripts was induced less than 10-fold, and was generally not significantly different between the young and mature culm internodes. However, *LEA *and *dehydrin *transcripts were dramatically induced by water stress, up to 100- and 1000-fold respectively, in both I4-5 and the lowest internodes. Differences in gene expression under water stress between immature and mature culms could be related to the differences in the water content in the two different tissues. The moisture content in I4-5 dropped much more over the 15 days of water stress, from 90% to 79%, compared to the lowest internodes where the moisture content remained stable at approximately 70% over the 15 days (Table 4). Therefore, even in the absence of a change in moisture and sugar content in the lowest internodes, a mechanism inducing expression of abiotic stress-related genes was in operation. This mechanism responded to water deficit stress independently of sucrose accumulation.

#### Amino acid content of sugarcane tissue under water deficit

**Figure 3:**
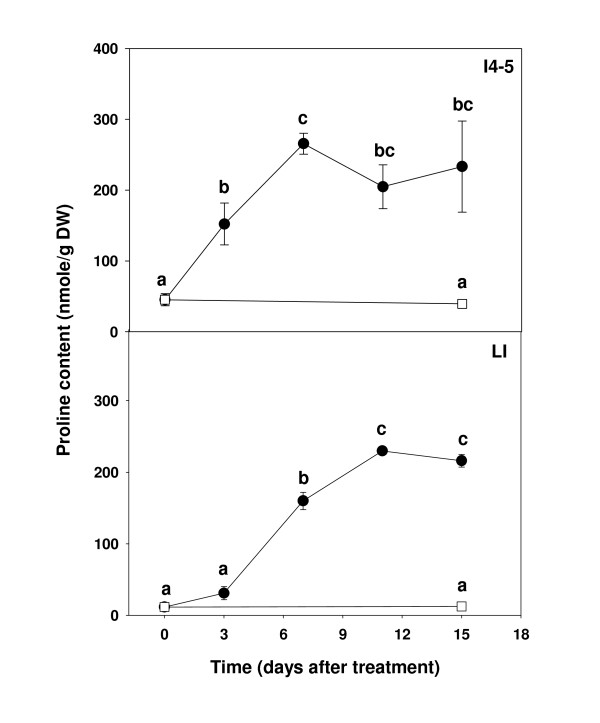
**Proline accumulation in response to water deficit**. Proline content in Q117 culm internodes 4-5 (I4-5) and the lowest internodes (LI) under water stress (black circle) and control (white square). Error bars indicate standard error of the mean (n = 3). Time points with the same letters are not significantly different based on LSD test (P ≤ 0.05) from one-way analysis of variance.

The levels of almost all amino acids increased after 15 days of water stress when compared with those of control plants (Table 5). Proline increased after three days of stress treatment relative to that of the T_0 _sample and continued to increase until 15 days of treatment in both the I4-5 and the lowest internodes (Figure 3). However, there were no changes in proline content in the well-watered control plants after 15 days. Although the proline content increased dramatically in the I4-5 and the lowest internodes in water deficit stressed plants, it only reached concentrations equivalent of 54-65 nmole/g FW. Therefore, proline was far from being the most abundant or most highly induced amino acid in the water-stressed samples. Increasing levels of all amino acids were measured mostly after three or seven days of the water deficit stress (data not shown). Asparagine and phenylalanine levels increased greatly after 15 days of water stress in both young and mature culm internodes. The most abundant amino acid after water deficit stress was asparagine, which increased over 20-fold, to levels equivalent to ~800 nmoles/g FW in both the I4-5 and LI samples. However, the content of some amino acids, e.g. aspartic acid and glutamic acid, appeared to increase at early stages of stress (data not shown) but subsequently decreased after 15 days of stress. Glutamic acid content was significantly lower after 15 days stress in both internodes.

**Table 5:** Free amino acid content in internodes 4 and 5 (I4-5) and the lowest internode (LI) taken from sugarcane cultivar Q117 15 days after imposition of water deficit.

	**Amino acid concentration (nmoles g**^**-1 **^**dry weight)**			
	
	I 4-5		LI	
	
Amino acid	Control	water deficit	Control	water deficit
His	18.63 ± 4.00^1^	179.12 ± 7.73*	6.32 ± 0.53	108.26 ± 1.52*
Arg	34.18 ± 2.03	117.52 ± 7.73*	12.06 ± 0.29	71.14 ± 5.32*
Asn	214.28 ± 14.18	4084.42 ± 317.28*	85.16 ± 2.57	2599.75 ± 143.49*
Ser	154.89 ± 25.48	758.93 ± 165.10*	46.95 ± 2.20	407.78 ± 9.71*
Gln	291.81 ± 26.48	253.39 ± 60.56	118.06 ± 8.06	364.07 ± 22.32*
Gly	89.41 ± 14.27	69.75 ± 7.58*	49.54 ± 2.26	68.56 ± 1.41*
Asp	227.72 ± 13.33	68.49 ± 14.06*	114.62 ± 4.96	111.15 ± 7.01
Glu	326.76 ± 9.10	63.29 ± 11.60*	161.97 ± 5.12	95.46 ± 2.43*
Thr	69.50 ± 6.14	323.55 ± 51.77*	23.70 ± 0.96	204.38. ± 6.96*
Ala	264.77 ± 20.88	415.80 ± 100.92*	112.68 ± 6.31	561.32 ± 4.56*
Pro	39.44 ± 4.62	233.27 ± 64.31*	12.46 ± 0.02	215.98 ± 8.75*
Tyr	78.86 ± 8.43	650.95 ± 23.51*	21.85 ± 0.16	105.72 ± 4.30*
Val	52.16 ± 4.45	235.96 ± 39.93*	18.18 ± 0.78	221.36 ± 2.00*
Met	10.21 ± 0.80	101.52 ± 21.45*	4.43 ± 1.13	72.30 ± 4.68*
Lys	36.44 ± 2.57	9.72 ± 3.26	12.08 ± 0.77	24.76 ± 1.05*
Ile	35.06 ± 3.57	220.21 ± 30.65*	12.33 ± 1.08	211.29 ± 3.35*
Leu	32.87 ± 3.81	191.23 ± 30.89*	9.90 ± 0.55	185.54 ± 3.17*
Phe	16.13 ± 2.23	484.31 ± 32.59*	5.64 ± 0.07	150.73 ± 7.99*

^1^Data are presented as mean values ± standard error of the mean (n = 3) * indicate significantly different concentration of stressed plants compared to control (Student t-test P ≤ 0.05).

## Discussion

It has been postulated that the accumulation of sucrose to a high concentration in sugarcane culm tissue may cause stress in the storage as well as non-storage cells due to the high solute concentrations in storage cells, and associated osmotic gradients between culm cell types [4]. Therefore, sugarcane culm cells are likely to have some adaptive mechanisms to protect and maintain their metabolism. A potential adaption is that stress tolerance mechanisms that facilitate cellular function under high solute load may be activated in sugarcane culms. These may be similar to those activated during other stresses that also lead to reduced osmotic potential such as water deficit stress. Large scale gene expression profiling has provided evidence that many transcripts with functions related to abiotic-stress tolerance and water deficit stress were abundant in internodes with a higher sucrose content [7-9]. However, as no expression studies in sugarcane have yet been able to assay all of the genes present in sugarcane, additional genes not present in all of these earlier studies, were also chosen.

This study has found that the expression of a number of genes involved in abiotic stress responses showed significant correlative relationships with sucrose content in sugarcane, not only across various culm tissues, but also across the mature culms of diverse *Saccharum *genotypes. Some transcripts that showed a positive correlation with sucrose content encoded predicted proteins with functions in the biosynthesis of proline (OAT) and asparagine (AS) and sugar transport (PST5). A negative correlation of expression with sucrose content across genotypes was also demonstrated for genes encoding an enzyme in an alternative proline biosynthetic pathway (P5CS) and the bZIP transcription factor TF1 which may have a regulatory function.

Expression levels of the putative sugar transporter PST5 showed a positive correlation with sucrose content both down the culm tissues and across diverse genotypes. The PST5 sequence is homologous to sugar transporter-like proteins from *Arabidopsis *that are a part of the ERD-6 (Early Responsive to Dehydration) group of transporters [20,21]. The ERD genes were induced in *Arabidopsis *by drought treatment and ERD-6 responds to both water deficit and cold. The PST5 gene appeared to be induced weakly by water deficit in sugarcane. The transporter encoded by PST5 has recently been localised to the tonoplast and may play a role in remobilisation of sugars from the vacuole [22]. Since sugar transport is a key component of current models for sugar accumulation in sugarcane culm tissue [1], the correlation of *PST5 *gene expression with sucrose content should stimulate interest in further functional analysis of a possible rate limiting role for this transporter in the sugar accumulation process.

Ten groups of bZIP transcription factors have been identified in *Arabidopsis *[23]. They have been demonstrated to have roles in biotic and abiotic stress responses, as well as plant development [23]. The bZIP transcription factor gene *TF1 *trended to higher expression in older culm tissue of Q117, but when tested across diverse genotypes, it was negatively correlated with final sucrose content. This suggests that the increased level of expression of *TF1 *reached in mature internodes may either be required for increased regulation of sucrose accumulation or be a response to it. The bZIP transcription factor with the closest homology to *TFI *is the OCS binding factor 3.1 from maize [24] and, like the most similar bZIP transcription factor in *Arabidopsis *(AtTGA6), it has been linked to defence mechanisms to counter biotic stress [25]. Some bZIP transcription factors are regulated by sugar levels in other plants, such as AtbZIP11 of *Arabidopsis*, which is repressed by sucrose, but also co-regulates the expression of asparagine synthetase 1 and proline dehydrogenase 2, linking sugar content with asparagine and proline metabolism [26]. However, AtbZIP11 is in the sequence group S of bZIP transcription factors [23] while *TF1 *is most homologous to members of group D [23]. In sugarcane, Gupta et al. [27] showed 12-fold induction of a bZIP transcription factor after leaves were treated with 400 mm mannitol for nine hours, suggesting a role in osmotic stress tolerance. Again, this bZIP protein was quite different to TF1, belonging to group G [23] and herein *TF1 *was only moderately induced during the osmotic stress caused by water deficit. Functional analysis of *TF1 *would be required in transgenic sugarcane to test the role in sucrose accumulation and any role in cross-regulation of amino acid metabolism.

The accumulation of particular amino acids is one of the responses of plant cells to osmotic stress. Our results showed transcripts predicted to encode proteins involved in proline metabolism (OAT, Pox, P5CS) appeared to be up-regulated in the mature culm. The main pathway for proline synthesis is believed to be from glutamate, which is directly converted to glutamic semialdehyde (GSA) by the enzyme P5CS (pyrroline-5-carboxylate synthetase), with GSA being then converted to P5C (pyrroline-5-carboxylate) by spontaneous cyclization. P5C is then reduced to proline by P5C reductase (P5CR) [28,29]. *OAT *encodes the enzyme in a secondary pathway of proline biosynthesis which converts arginine to ornithine and then via several intermediate steps to proline. In our data describing the between-genotype comparison, there was a significant positive correlation (P = 0.03) between *P5CS *expression and the free proline content, while *OAT *expression was correlated negatively with proline content. These correlations are consistent with proline synthesis being predominantly genetically regulated through *P5CS *expression and synthesised via the glutamate pathway rather than via ornithine.

Proline is a well known compatible solute as well as an antioxidant and osmoprotectant [30], and can accumulate to high concentrations in plant cells under osmotic stress [28,11]. In tobacco, proline accumulated from 0.69 to 26.1 μmoles/g FW after 10 days of drought treatment [13], while in rice, proline concentration increased about five-fold (from 0.5 to 2.3 μmol/g FW) under salt stress [31]. The analysis of free proline in the culm also indicated a significant negative correlation with sucrose content, which was consistent with a similar negative correlation of expression of the major biosynthetic gene *P5CS*. The decrease in proline levels in culm tissues with higher sucrose content clearly contradicts the possibility that proline plays a role in osmoprotection associated with sucrose accumulation in the culm. Previous studies have suggested that proline is one of the major free amino acid in sugarcane culms [17]. However, our refined analysis of this amino acid indicates that it accumulated at low levels in mature sugarcane culms (<5 nmoles/g FW), and even under water deficit stress it increased only to 54-65 nmoles/g FW, which is a much lower proline concentration than detected in other plant species undergoing osmotic stress. Therefore, our results do not support a role of proline as an osmoprotectant during sucrose accumulation and question whether it has a significant role even under water deficit stress. A study of proline biosynthesis in leaves of control and transgenic sugarcane plants expressing a heterologous *P5CS *gene has also questioned a potential role for proline in osmotic adjustment under water deficit and alternatively suggested a role as an antioxidant, where lower concentrations may be effective [30].

Levels of most of the free amino acids measured were elevated under water stress both in young and mature parts of the culm, resulting in an approximately seven-fold increase in the total amino acid content. The expression of the *AS *gene, encoding a transaminase responsible for the synthesis of asparagine from aspartate and glutamine [32], was positively correlated with sucrose accumulation in developing culms and across diverse genotypes. However, there was no clear relationship between the levels of free asparagine with sucrose content. Asparagine accumulated to high levels in plants exposed to water deficit (~800 nmoles/g FW), suggesting that this amide may have some role in adaptation to water deficit stress in sugarcane. These differences in asparagine responses suggest metabolic differences exist between the cellular adaptation mechanisms associated with high solute loads resulting from sucrose accumulation and the adaptation response to water deficit stress.

Genes that were highly induced under water deficit were not correlated with sucrose content in the culm across different genotypes. The genes encoding protein chaperones, LEA and dehydrin, were dramatically induced by water deficit by more than a 100-fold, with greater fold induction in the immature culm when compared to the mature culm. These types of chaperones play a role in the protection of proteins from degradation and the action of proteinases [33]. The *LEA *gene family was first identified as genes induced in seeds during maturation and desiccation [34], while in vegetative tissues, LEA proteins are induced by osmotic stress and ABA [35]. In our study, *LEA *and *dehydrin *were elevated in the mature internodes of Q117 when compared to the other tissues. These genes were not reported as being up-regulated by Rodrigues et al. [36], when comparing well-watered and droughted young sugarcane plants but this is because clones for these genes were not represented on their array. A clone encoding dehydrin (SCQGLR1085F11.g, part of TC114145 used in our study) was included in the array used by Papini-Terzi et al. [9] and they observed that it was more highly expressed in more mature internodes, but less well-expressed in high brix plants when compared to low brix plants. When the same clone was compared in plants subjected to water deficit by Roca et al. [10], expression was elevated in above ground tissue after 72 hours. In our study, there was no significant difference in the expression of this dehydrin gene in mature internodes of genotypes with varying sucrose content but it was also strongly expressed after water deficit.

Other genes whose transcript expression levels were significantly positively or negatively correlated with sucrose content in the culm, were only slightly induced by water stress. Transcripts of genes associated with amino acid metabolism, such as *P5CS*, *OAT *and *AS*, were induced more than 10-fold during water stress, especially in the immature culm tissue. However, the expression of *Pox*, a gene encoding an enzyme that hydrolyses proline to P5C, was extremely suppressed in the mature culm under water deficit. This probably explains the increase in proline content under water deficit stress, both in immature and mature culms. Conversely, the expression levels of the bZIP *TF1 *transcription factor and the putative sugar transporter *PST5 *were only slightly increased in response to the water stress treatment, yet their expression patterns correlated with the level of sucrose accumulation across a range of genotypes.

## Conclusions

Whilst we have not assessed the expression of all of the genes of sugarcane, correlative experimental evidence suggests that the expression of the genes related to the molecular processes studied involving osmoprotectants, water and ion movement and chaperones may not limit sucrose accumulation in the sugarcane culm. However, a stress-related transcription factor and sugar transporter may play a role in sucrose accumulation. We also found that protection against any stress caused by sucrose accumulation appears to use different mechanisms to those used to protect from stress induced by water deficit. Sucrose accumulation is a complex process and it is likely that there are other mechanisms beyond those explored herein that act to limit sucrose accumulation.

## Methods

### Plant materials

#### Stress-related gene expression and sucrose content in cultivar Q117 tissue at different developmental stages

Sugarcane cultivar Q117 was grown in a glasshouse, at Indooroopilly, Brisbane (27°30' 48"S; 153°59'48"E). Culm pieces with one bud (single eye setts) were planted in plastic trays containing peat (Searles Peat 80+, J.C. & A.T. Searle Pty Ltd, Queensland, Australia) on 10 October 2003. Three plants per pot were transferred to 30 cm diameter by 30 cm deep plastic pots containing peat, on 7 November 2003. Cooling and heating was applied when temperatures were above 32°C and below 22°C respectively. Plants were watered to the capacity of the pots by an automatic system twice a day. Fertiliser was applied once a month using liquid foliar nutrient fertilizer (Wuxal, N 9.9: P 4.3: K 6.2; Aglukon, AgNova Technologies Pty Ltd, Victoria, Australia) at a rate of 150 mL per pot (30 mL supplier concentration in 4 L of water) and 10 g of slow release fertilizer (Apex Gold with polyon, N 17: P 7.3: K 14.1; Simplot ASIA Corp. Lathrop, CA, USA). Plants from three pots were harvested in April 2004 and treated as replicates. The lamina of the last fully expanded leaves, meristem to internode 2 (M-I2), internodes 4-5 (I4-5), internodes 7-8 (I7-8), and internodes 13-14 (I13-14) or the lowest internodes were cut from the main stalk of each plant. Tissues were pooled from the plants within a pot. Samples were frozen in liquid nitrogen and stored at -80°Cfor analyses of sugars and gene expression. In all experiments, internodes were numbered from the top of the culm towards the base as described by Moore [37] i.e. the first internode is the one immediately below the node to which the last fully expanded leaf subtends.

#### Gene expression, sugar and amino acid content in sugarcane genotypes varying in sucrose content

Thirteen genotypes comprising the commercial cultivars Q28, Q117, Q124, Q165, and Q200^A ^the progenitor species of commercial cultivars *S. officinarum *clones Badilla, IJ76-237, IJ76-567, NG51-99, NG77-98, and *S. spontaneum *clones Mandalay and SES106, and an *Erianthus arundinaceus *clone were planted in peat on 15 February 2005 in a glasshouse at Indooroopilly, Brisbane. Cooling was initiated when temperatures were above 31°C and heating applied when the temperature fell below 24°C. Single eye setts were transferred to 30 cm diameter pots filled with peat on 9 March 2005 with three plants per pot. Three pots of each genotype were arranged in a completely randomised design and maintained under the same conditions. Clones were harvested one replicate per day, on 29-31 August 2005 to reduce diurnal effects. Samples of the tissue of internodes 13 and 14 (I13-14) were harvested as previously described and cut into approximately 0.5 cm^3 ^pieces and frozen in liquid nitrogen as quickly as possible. Samples were stored at -80°C until analysed.

#### Gene expression, sugar and amino acid content in sugarcane Q117 with water deficit stress

Plants on which water deficit stress were imposed were grown in pots in a glasshouse at St. Lucia, Brisbane (27°29'53" S; 153°00'37" E), from November 2004 to April 2005. Conditions inside the glasshouse, which had ambient lighting, were set at 31°C/22°C for day and night temperatures, respectively. Relative humidity was set at 55% during the day and 71% at night. Single eye setts were propagated first in plastic trays from 22 November 2004 then transferred to 30 cm diameter plastic pots with 3 buds in each pot. Fertilisers were applied as described above. Stress was induced by withholding water supply starting from 12 April 2005 when the plants had grown 13-14 internodes and was continued for 15 days until 27 April 2005. The pots given the stress treatment were chosen randomly and the control plants were watered until harvested. The experimental plan involved sampling water stressed plants at four different times and non-stressed or control plants at two different times at the beginning (T_0_) and end of the treatment (C_15_). Water stressed plants were sampled after 3 days (T_3_), 7 days (T_7_), 11 days (T_11_), and 15 days (T_15_). The three stalks in each pot were combined when they were sampled. Samples of tissues were taken from the last fully expanded leaf (LFE), M-I2, I4-5, I7-8_, _and the lowest internodes (I13-15). All samples were cut into approximately 1 cm^3 ^pieces and frozen in liquid nitrogen as quickly as possible then stored at -80°C until analysed.

### Selection of genes and primer design

Fifty one genes associated with osmotic stress in the literature, have been collated and their putative functions and primer sequences are listed in additional file 2. Seventeen genes up-regulated during culm maturation of sugarcane that were identified from microarray analysis [6-8] were selected. As all arrays being used to assay gene expression from sugarcane contained only a relatively small sub-set of genes, sugarcane homologues of other genes identified from the literature [11,23,24,28,35,38-55] reported to be involved in stress tolerance were also included in the study. The genes and their predicted proteins were grouped into functional categories with roles in: osmoprotection (e.g. biosynthetic enzymes for polyamines, amino acids, sugars and polyols), water and ion movement (e.g. aquaporins, ion transporters), chaperones (e.g. *Hsp80*, *LEA*, *DnaJ*), sugar transporters (e.g. *ShSUT1, PST2a, PST7*), transcriptional factors (e.g. *DREB*-like gene, *HvDRF*, *TF1*,), and other stress-related genes (e.g. *osmotin*, *expansin*, *lipoxygenase*). A comparison of nucleotide sequences from several plants but mainly rice and maize coding sequences, and sugarcane ESTs was used as a basis for the design of primers specific to the target genes. Primers for most of the transcription factor genes (*THB43-11, TW26b-10, TAP24F-4, TF1, HvDRF1, TZP16b-17, TWC1, TM89-33*, and *TM11b*) were designed on the basis of sequence comparison between sugarcane ESTs and wheat transcription factors that showed up-regulated expression in wheat under drought stress (DNA sequences of the transcription factors in wheat were kindly provided from Dr Gang-Ping Xue, CSIRO). The homology of selected gene sequences to sugarcane genes was examined using BLASTN searches [56] of the dbEST and non-redundant nucleotide databases at NCBI http://www.ncbi.nlm.nih.gov/ limited to *Saccharum sp *for dbEST, as described by Iskandar et al. [16], and the Sugarcane Gene Index 3.0 http://compbio.dfci.harvard.edu/tgi/cgi-bin/tgi/gimain.pl?gudb=s_officinarum. Primers for each gene were tested for specificity by standard PCR, with those yielding positive results further tested by RT-qPCR [15]. All primers were designed using Primer Express software (version 1.5, Applied Biosystems, USA) set to an annealing temperature between 58-60°C with an amplicon size of 100 to 150 bp. All primers were synthesized by GeneWorks Pty. Ltd., Australia.

### RNA and cDNA Preparation

Sugarcane tissues were ground using a mill (Retsch Mixer Mill, #MM300). Samples were placed in 25 mL stainless steel grinding jars pre-cooled in liquid N_2_, together with one 20 mm stainless grinding ball per jar (MEP Instruments Pty Ltd, North Ryde, Australia). Total RNA was extracted from ground tissue using a CsCl pad method and synthesis of cDNA were carried out as described in Iskandar et al. [16].

### Real time quantitative PCR after reverse transcriptase of RNA (RT-qPCR)

Real time quantitative PCR (RT-qPCR) was performed as described in Iskandar et al. [16] with the modifications detailed below. For analysis of gene expression of the different tissues of Q117, reactions were conducted in an ABI Prism^® ^7000 Sequence Detection System (Applied Biosystems). For the experiments of gene expression in sugarcane genotypes varying in sugar accumulation and sugarcane imposed with water deficit stress, RT-qPCR was performed in an ABI Prism^® ^7900HT Fast Real-Time PCR System (Applied Biosystems). The total reaction volume was 10 μL, containing 2 μL of template cDNA, 5 μL of 2X SYBR green master mix (Applied Biosystem), 2 μL of primer mix (1 μM) and 1 μL of water. All samples were amplified in triplicate assays using the following conditions: 50°C for 2 min and 95°C for 10 min, for 1 cycle, followed by 40 cycles of 95°C for 15 s and 59°C for 1 min. The dissociation stage was performed at 95°C for 2 min, followed by 60°C for 15 s and then at 95°C for another 15 s. A fluorescence threshold was set manually to a ΔRn of 0.2 on the log fluorescence scale to determine the Ct value, and the baseline was set to the default between cycle 3 to 15. Data were analysed using SDS 2.2 software (Applied Biosystems) and Microsoft Excel. RT-qPCR assays were used to measure transcript abundance of the target genes (listed in additional file 2), using glyceraldehyde-6-phosphate dehydrogenase (GAPDH) as a reference gene [16].

### Sugar analysis

Sugars were extracted by incubating up to 2 g of ground tissue (ground as for RNA extraction) with 9.9 mL of water in a water bath at 95°C for 15 min then overnight at 70°C. The extract was transferred to a new tube and the tissue extracted a second time with 9.9 mL water at 70°C overnight. Following the second extraction, the two extracts were pooled and kept frozen at -20°C until analysis as described by Glassop et al. [57]. Glucose, fructose and sucrose were separated by High Performance Liquid Chromatography (HPLC), detected by pulsed amperometric detection (Waters 464; Waters) as described by Albertson and Grof [58] and quantified by Empower software http://www.waters.com using standard curves calculated from external standards that were processed for each group of ten experimental samples [57].

### Amino acid analysis

Amino acids were analysed using HPLC and Ultra High Performance Liquid Chromatography (UPLC). Samples for amino acid analysis were prepared by two different extraction methods; hot water extraction for HPLC and methanol extraction for UPLC. The HPLC analysis utilised a sub-sample of the extracts prepared for sugar analysis. A concentrate was made from 200-500 μL of each sample by centrifugation under vacuum (Hetovac, Heto, Scandinavia) and re-dissolving the solid residue in 50 μL of MQ water. A 30 μL aliquot of each sample was derivatised using Waters AccQ.Tag methodology following the protocol in the instruction manual of Waters Inc. The HPLC system consisted of an autosampler (Waters 717 plus), a multisolvent delivery system (Waters 600E), a fluorescence detector (Waters 2475), and a column (Waters AccQ.Tag amino acid 4 μm particle size, silica base bonded C18, 44 μm sieve size; 3.9 x 150 mm). The separation time was 45 min using a single pump gradient system with excitation at 250 nm and detection of amino acids at 395 nm. Mobile phases used were (A) acetate-phosphate buffer (Waters AccQ.Tag Eluent A), (B) 100% Acetonitrile and (C) Milli-Q water. The amino acid standard was prepared with a concentration of 2.5 μM/mL of Asp, Ser, Glu, Gly, His, Arg, Thr, Ala, Pro, Tyr, Val, Met, Lys, Cys, Ile, Leu and Phe, and diluted to make a range of 50-500 pmol within 5 μL of injection volume.

For UPLC analysis, methanol extraction was carried out by mixing around 0.2 g of ground sample with 1 mL cold 20% methanol (approximately 5v/w ratio), vortexing and incubation at -20°C overnight. After centrifugation at 10,000 g for 5 min, 500 μL of the supernatant was concentrated by centrifugation under vacuum and the pellet was resuspended in 50 μL of 20% methanol. A 20 μL aliquot was derivatised by the following methods in a total volume of 200 μL by AccQ.Tag following the protocol from Waters. Samples were derivatised using 6-aminoquinolyl-*N*-hydroxysuccinimidyl carbamate which was prepared according to Cohen and Michaud [59], and buffered using 0.2 M borate buffer (sodium borate, sodium carbonate and sodium bicarbonate) pH 8.8. Samples were filtered using a 0.2 micron AcroPrep 96 filter plate. Separation of amino acids was performed on a C18 column (Waters Acquity UPLC BEH C18). Mobile phases used were 0.1% Di-N-Butylamine, 0.2% acetic acid as solvent A and 55% acetonitrile as solvent B. The gradient for sample separation was initially 100% A, after 0.1 min 96% A and 4% B, after 2 min 92.5% A and 7.5% B, after 7 min 60% A and 40% B, after 7.5 min 40% A and 60% B, after 8.5 min 40% A and 60% B, and finally after 8.6 min 100% A, at a flow rate of 0.4 mL min^-1 ^throughout. Derivatised amino acid residues were detected by absorption at 254 nm. External standards were made using the Waters physiological amino acid standard with addition of Asn, Gln, GABA and Trp (Sigma). This standard was diluted to make a standard curve with concentrations ranging from 20 to 500 μM. The standards were run after every 12 samples.

### Physiological measurements of water stress

To quantify the level of water stress, relative water content (RWC), stomatal conductance, and photosynthetic rate were measured on the last fully expanded leaf. RWC was determined using the relative turgidity method described by Barrs and Weatherly [60]. Stomatal conductance and photosynthetic rate were measured on the middle part of the leaf blade outside the midrib, using a portable photosynthesis system (Li-6400, Li-COR Inc., Lincoln, Nebraska, USA). Measurements were made on day 0 (at the start of the stress treatment) and 3, 7, 11 and 15 days thereafter. For control plants, measurements were made at days 0 and 15 days. Measurements were taken using the internal Red/Blue light source (6400-02B LED Light Source) with the intensity of 2000 μmol m^-2^s^-1^, and CO_2 _mini cartridges were used as the CO_2 _source supplied at 400 μmol s^-1^.

### Statistical analysis

Statistical analysis (ANOVA and correlation test) of the data was conducted with GENSTAT (VSN International Ltd. Herts, UK) and by GeneSpring (Agilent Technologies, CA, USA).

## Authors' contributions

HMI carried out the molecular analyses, physiology experiments and drafted the manuscript. REC participated in the design of the study and provided expert advice on molecular techniques. ATF participated in the design and execution of the amino analysis. SS participated in the design of the amino acid analyses. JX carried out the sugar measurements. DJM participated in the design and coordination of the study. JMM conceived the study and participated in its design and helped draft the manuscript. GDB participated in the design of the study, coordinated the study and helped draft the manuscript. All authors read and approved the final manuscript.

## Supplementary Material

Additional file 1 **Amino acid concentration in mature internodes of 13 genotypes**. The amino acid concentration of 20 amino acids from the lowest internode of 13 genotypes is presented, along with the sucrose content. Click here for file

Additional file 2 **Primers for genes assayed**. The group of functional genes that the gene is part of, the full and abbreviated names, literature source of the gene, sequence identity number, sequence identity of best match, score and E value and forward and reverse primers used to amplify the genes in the PCR assay are presented in the table. Click here for file
