# Supplementary material for: Identification of drought-response genes and a study of their expression during sucrose accumulation and water deficit in sugarcane culms
Source: BMC Plant Biol. 2011 Jan 13;11:12. doi: 10.1186/1471-2229-11-12 (PMC3030532; doi:10.1186/1471-2229-11-12)
Supplement: Additional file 1 — Amino acid concentration in mature internodes of 13 genotypes. The amino acid concentration of 20 amino acids from the lowest internode of 13 genotypes is presented, along with the sucrose content. [file 1471-2229-11-12-S1.DOC]

Additional file 1 Concentration of individual amino acids (nmoles/g FW) in the lowest internodes of 13 different sugarcane genotypes measured by UPLC.

| Amino acid | SES- 106^1^  5.97 | Erianthus  16.8 | Mandalay  40.6 | Badilla  88.5 | Q28  97.8 | Q165  115.0 | IJ 76-237  119.3 | NG 51-99  120.7 | IJ 76-567  124.7 | NG 77-98  130.6 | Q200  132.5 | Q124  141.3 | Q117  143.2 | Correlation  (R value) |
| --- | --- | --- | --- | --- | --- | --- | --- | --- | --- | --- | --- | --- | --- | --- |
| His | 10.4 | 8.3 | 5.7 | 15.0 | 23.1 | 14.3 | 6.1 | 15.1 | 11.2 | 12.9 | 8.3 | 10.8 | 6.5 | 0.043 |
| Arg | 23.3 | 7.6 | 17.0 | 11.9 | 15.1 | 16.4 | 7.0 | 27.1 | 13.6 | 21.4 | 11.4 | 14.2 | 8.2 | -0.050 |
| Asn | 152.0 | 59.1 | 80.6 | 2590 | 3267 | 2621 | 355.1 | 1800 | 705.5 | 2903 | 2523 | 1033 | 262.2 | 0.406 |
| Ser | 80.7 | 128.1 | 70.1 | 179.4 | 320.7 | 100.0 | 95.2 | 226.2 | 121.9 | 208.6 | 96.1 | 97.4 | 60.3 | 0.048 |
| Gln | 182.7 | 183.6 | 150.1 | 1340 | 1126 | 449.6 | 427.5 | 482.8 | 1064 | 595.4 | 450.4 | 506.0 | 217.0 | 0.239 |
| Gly | 40.1 | 19.4 | 26.8 | 55.7 | 64.7 | 35.6 | 30.0 | 46.7 | 36.8 | 39.3 | 23.9 | 26.7 | 14.2 | -0.095 |
| Asp | 113.8 | 284.9 | 58.4 | 83.9 | 62.1 | 101.8 | 93.8 | 142.7 | 168.6 | 94.3 | 100.5 | 132.6 | 127.3 | -0.420 |
| Glu | 142.5 | 345.0 | 98.6 | 56.8 | 39.8 | 55.7 | 93.5 | 119.0 | 190.8 | 62.8 | 89.4 | 124.7 | 79.9 | -0.562 |
| Thr | 28.4 | 42.6 | 24.4 | 113.7 | 93.4 | 21.6 | 32.4 | 61.1 | 46.6 | 51.4 | 34.2 | 40.3 | 22.1 | -0.040 |
| Ala | 117.3 | 132.9 | 71.3 | 146.9 | 607.3 | 72.2 | 114.1 | 291.9 | 88.9 | 205.8 | 75.1 | 94.7 | 67.2 | -0.042 |
| GABA | 103.3 | 110.0 | 119.2 | 61.0 | 61.1 | 99.5 | 93.0 | 156.5 | 81.2 | 97.1 | 77.0 | 87.1 | 98.2 | -0.099 |
| Pro | 14.1 | 15.0 | 11.3 | 12.0 | 9.8 | 9.2 | 7.1 | 12.9 | 11.1 | 9.0 | 6.9 | 8.3 | 6.3 | -0.824** |
| Tyr | 27.2 | 21.3 | 23.6 | 19.8 | 39.9 | 20.9 | 12.8 | 25.7 | 16.1 | 16.4 | 20.8 | 14.9 | 15.3 | -0.381 |
| Val | 31.2 | 30.4 | 27.0 | 51.2 | 102.4 | 51.8 | 41.9 | 75.7 | 78.2 | 57.7 | 56.9 | 48.8 | 28.4 | 0.361 |
| Met | 36.6 | 57.2 | ND | 9.6 | 25.9 | 7.4 | ND | 26.5 | 55.1 | 12.2 | 11.5 | 57.5 | 46.5 | -0.183 |
| Lys | 34.6 | 21.3 | 24.9 | 7.7 | 13.1 | 10.0 | 13.2 | 19.9 | 20.5 | 8.0 | 16.3 | 16.9 | 19.3 | -0.565 |
| Ile | 19.3 | 19.1 | 14.9 | 16.7 | 28.8 | 15.5 | 15.5 | 25.8 | 24.3 | 14.6 | 12.6 | 16.5 | 9.4 | -0.238 |
| Leu | 29.2 | 25.3 | 21.6 | 13.0 | 17.0 | 11.8 | 11.8 | 16.5 | 19.9 | 11.0 | 12.6 | 14.9 | 12.0 | -0.860** |
| Phe | 17.7 | 11.4 | 16.4 | 7.6 | 30.3 | 10.8 | 6.6 | 15.4 | 9.9 | 10.3 | 12.4 | 11.3 | 8.4 | -0.238 |
| Trp | 11.1 | 14.5 | 10.7 | 12.4 | 40.8 | 10.1 | 6.2 | 19.3 | 21.7 | 10.8 | 11.2 | 16.0 | 12.3 | 0.049 |

^1^The genotype names are indicated and numbers below the genotypes name indicating their sucrose content (mg suc/g FW).

**A significant correlation with sucrose concentration (R) is indicated with asterisks (** = P≤ 0.01). ND = not detected
