# Supplementary material for: Identification of drought-response genes and a study of their expression during sucrose accumulation and water deficit in sugarcane culms
Source: BMC Plant Biol. 2011 Jan 13;11:12. doi: 10.1186/1471-2229-11-12 (PMC3030532; doi:10.1186/1471-2229-11-12)
Supplement: Additional file 2 — Primers for genes assayed. The group of functional genes that the gene is part of, the full and abbreviated names, literature source of the gene, sequence identity number, sequence identity of best match, score and E value and forward and reverse primers used to amplify the genes in the PCR assay are presented in the table. [file 1471-2229-11-12-S2.DOC]

### Additional file 2 Primers for genes assayed

| Group | Full name of gene | Name of gene in the text  (Source) | Sequence ID^*^ | Sequence ID of best match (not self) ^#^ | Max score of best match | E value of best match | Primer sequences |
| --- | --- | --- | --- | --- | --- | --- | --- |
| Osmoprotection – Polyamines | S-adenosyl methionine decarboxylase | *SAM-dc*  [7,8] | TC117892 | GQ380442 | 1485 | 0.0 | F: CATGATGTATGAAGCTCCCCTTG  R: TCTTCGCGCAGTTGGAGTAAG |
|  | S-adenosyl methionine synthetase (SAM synthase) | *SAM-synt*  [7,8] | TC144476 | BT016781 | 1680 | 0.0 | F: TCCAGGACACCACCCAGATC  R: GGGTTCTCGAGGTGACACATC |
|  | Arginine decarboxylase | *ADC*  [8,38] | TC145364 | BT085866 | 1613 | 0.0 | F: CTCTACAACGTGGACGGCTG  R: AGGTCGATCTCCTGGCCTG |
|  | Agmatine deaminase | *ADI*  [38] | TC136583 | XM_002446593 | 1440 | 0.0 | F: CGATGATGAGAAGGACCCTCAG  R: ATCTTCACCACTTCGAGCTGC |
|  | Spermidine synthase | *SPDS*  [39] | TC140026 | BT039624 | 702 | 0.0 | F: TGATGCCGTGTGGATCTCTG  R: TGTTGATTTCCAGCACCCTG |
| Osmoprotection - Amino acids | Pyrroline-5-carboxylate synthetase | *P5CS*  [10,24] | TC112688 | EU113257 | 4529 | 0.0 | F: CCTGATGCCTTGGTCCAGA  R: TGCAATACTGTGTTTGATCTCATGG |
|  | Ornithine aminotransferase | *OAT*  [7] | TC128946 | XM_002441213 | 1260 | 0.0 | F: GTAAACCAGGCAAAGCGGATC  R: GATCACCAGGCATTTTGGAAG |
|  | Proline oxidase | *Pox*  [28] | TC119198 | XM_002467483 | 1016 | 0.0 | F: CGAGCGTGTGCATCAAGATC  R: GTCTTCCATGGCAGGTTGAAC |
|  | Proline Transporter | *ProT*  [28] | TC125141 | AY108640 | 957 | 0.0 | F: TCCCACTGACGTTTGTGCTC  R: AACCCAACAACATTCAGCCAG |
|  | Betaine aldehyde dehydrogenase | *BADH*  [40] | TC118507 | XM_002444312 | 1213 | 0.0 | F: GCTGCATGGGACATGGATG  R: CCATTGGAAGAGAAACTGGTGAG |
|  | Asparagine synthase | *AS*  [8] | TC116434 | EU953324 | 2535 | 0.0 | F: GCAGCAAGAAGCCGAGGAC  R: CCGGAGAAGCGATAGCATG |
| Osmoprotection - Sugars and Polyols | Trehalose phosphate synthase | *TPS*  [41] | TC120751 | XM_002452914 | 1653 | 0.0 | F: CCAACAGCAGCGTGAAGCT  R: TCCAGCATCTTGATCACGTCC |
|  | Trehalose phosphate phosphatase | *TPP*  [41] | TC134231 | BT060680 | 1159 | 0.0 | F: TCAGATGGGTCTCTCAGCGAG  R: CGCCTTCAAGGTACTTAGGGAG |
|  | Trehalase | *Tre*  [41] | TC131823 | DQ641513 | 1471 | 0.0 | F: CCGCTGTGGCTAAATGCATAC  R: TGCGGCATGAAGTAATCCAG |
|  | Mannitol dehydrogenase | *Mtl*  [42] | TC121521 | XM_002447037 | 1723 | 0.0 | F: GGTCAACACTTGCCAGTCCTG  R: GGTGACAGTGCCATCCCTG |
|  | Galactinol synthase | *Gols*  [43] | TC114091 | XM_002467909 | 2069 | 0.0 | F: AGTACAGGCCGATCCCCGAAC  R: GCAGTAGTGCACGGCCTTC |
| Water and ion movement | Plasma membrane intrinsic protein | *PIP*  [44,45] | TC127858 | XM_002454463 | 1894 | 0.0 | F: CACCGTCATGGGCGTCTC  R: GCAGTAGACGAGCGCGAAG |
|  | Tonoplast intrinsic protein | *TIP*  [44,45] | TC143602 | XM_002465814 | 1299 | 0.0 | F: GGCATGGCCTTCAGCAAG  R: GACACCGCCACGAACAGC |
|  | Na^+^/H^+^antiporter | *Hantiporter*  [8] | TC132075 | XM_002443629 | 1496 | 0.0 | F: TGGCAGGAAAGCGCTCTG  R: TCGTCGACCTTTCAGCTATGAG |
|  | Tonoplast H^+^PPase | *HPPase*  [8] | TC112793 | BT066389 | 4049 | 0.0 | F: AGCCAGCCCTGCACCTATAG  R: CAGAGAGGTGATGGCTCCAAG |
| Chaperone functions | DnaJ | *DnaJ*  [8] | TC125530 | XM_002466702 | 1591 | 0.0 | F: CTCTTTCGCGCAATGTCCTC  R: CGGATAGTGACTTTCATGCCTG |
|  | DnaK | *DnaK*  [8] | TC121466 | XM_002442308 | 1840 | 0.0 | F: ACAGTCCCTGCCTACTTCAACG  R: CCATAGGCGATAGCAGCAGC |
|  | dehydrin | *dehydrin*  [8] | TC114145 | XM_002439642 | 729 | 0.0 | F: ACCAGTACGGCAATCCAGTTG  R: CGGAGCGATGCAGGATG |
|  | Late embryogenic abundant | *LEA*  [35] | CA094376 | GQ494000 | 715 | 0.0 | F: TGTTTCTCCATCTCCCGAGTG  R: CATGGCAGGGTCTCTCAAGC |
|  | ABC transporter | *ABC transporter*  [35] | TC146958 | BT086091 | 1157 | 0.0 | F: ACCTTCCTCTCCGCGGTG  R: GATCCTGTCGCGGTTGATG |
|  | Hsp80 | *Hsp80*  [46] | TC113406 | XM_002444759 | 1142 | 0.0 | F: TGGAGATCAACCCGGAGAAC  R: CTCGAACAGCAGCATGACAAG |
| Transcription factors | DREB-like protein | *DREB* like protein  [47] | CA213057 | XM_002454494 | 767 | 0.0 | F: CGTGGACGACGACTGCTG  R: CACCCACTTGCCCCAGAC |
|  | TM 11b | *TM 11b*  (Gang Ping Xue, pers comm.) | TC114414 | BT039131 | 1232 | 0.0 | F: GCAGGGTCGATATGGTGACAG  R: CAACGACGTTCACCGAACAG |
|  | TZP 83-39 | *TF1*  (Gang Ping Xue, pers comm.; [24,23]) | HQ398871 | BT034456 | 1911 | 0.0 | F: GGGAGGACTGTTGCAGGTTG  R: TGGTTAGGTGGCTTCCGATC |
|  | TM 89-33 | *TM 89-33*  (Gang Ping Xue, pers comm.) | CA130879 | XM_002450307 | 789 | 0.0 | F: GAACGCAACCATGTCGGAG  R: GCGGTCACGGTCTCGAGTAC |
|  | THB43-11 | *THB43-11*  (Gang Ping Xue, pers comm.) | TC118541 | AK241438 | 848 | 0.0 | F: CGGCAGGAGCTAAAGCATG  R: AGGTAGAAGCAGTGTCGCCAG |
|  | HvDRF1 | *HvDRF1*  [48] | TC147038 | FJ805749 | 1173 | 0.0 | F: CACAATGTGGGTACCGTGGAG  R: AAAGGTACCCAGCCAGAGTCTG |
|  | TAP24F-4 | *TAP24F-4*  (Gang Ping Xue, pers comm.) | TC131346 | EU970244 | 1103 | 0.0 | F: GATCCGTTTATGCTGCTGCAG  R: GCCGTTCATGTCGTTGTTCAC |
|  | TZP16b-17 | *TZP16b-17*  (Gang Ping Xue, pers comm.) | TC134771 | XM_002439711 | 1745 | 0.0 | F: GAAAGTAACATCGCCACC  R: CCGTCTTGGCAAGATCATCAG |
|  | TWC1 | *TWC1*  (Gang Ping Xue, pers comm.) | TC129500 | XM_002462564 | 1604 | 0.0 | F: AAGCTCAAGGAGGGCGATG  R: GGCTGGTGAACGACTTGGAC |
|  | TW26b-10 | *TW26b-10*  (Gang Ping Xue, pers comm.) | TC125853 | BT054888 | 1294 | 0.0 | F: GATCCAAGGGTCTTCACCAATC  R: TGTTGCGTCGCTGTGGTTAG |
| Sugar transporters | PST2a | *PST2a*  [6] | AY165599 | GU066766 | 2493 | 0.0 | F: GTGCCCTGTTGGTTGGTATTG  R: TGCCACACCAGCTTGCTC |
|  | PST2b | *PST2b*  [6] | TC145400 | XM_002467535 | 1687 | 0.0 | F: GCTCCGGATCCAGAGAAGATC  R: ACCTAACGCGCTTCCAAGAG |
|  | PST3 | *PST3*  [6] | CA179545 | BT087015 | 572 | 5e-160 | F: TGCTCGCCTCCATGAACTC  R: TCTGCGTGTCCGTGATCTTG |
|  | PST4b | *PST4b*  [6] | CA117781 | XM_002460879 | 746 | 0.0 | F: CATCGAGCCCAGGAAGAAGAG  R: TCCACTCATCACCCCAATGTC |
|  | PST5 | *PST5 -* ([6] now *VMS1*) | HQ398872 | EU955778 | 2417 | 0.0 | F: CAAAGGCCAGGCTTCAAGAC  R: CACCATTTATCCCTCCGAGTTG |
|  | ShSUT1 | *ShSUT1*  (*PST6* - [6]) | AY780256 | GU812864 | 2654 | 0.0 | F: TCATCAGCCTTGCTGTCATTG  R: CCAGCGAGGACCATGATAGAG |
|  | PST7 | *PST7*  [6] | TC130402 | XM_002451900 | 1483 | 0.0 | F: GTGGCATTCTGTTGTTTGTCTTG  R: CATGCAGAGAGCCATAGCCTTAG |
| Other stress proteins | Osmotin | *Osmotin*  [49] | CA246318 | XM_002443345 | 839 | 0.0 | F: AGAACACCATGGCTGGCAC  R: CACAGGTTGTGGAGCGTCAG |
|  | Lipoxygenase | *Lipoxy*  [50] | TC126824 | XM_002458860 | 2266 | 0.0 | F: ACGCGGCCGTCAACTTC  R: TGCGCACGAACACCTTCTC |
|  | Expansin | *Expansin*  [51] | TC150814 | XM_002464900 | 829 | 0.0 | F: GAGCTTTAACGCTTCCGACTTC  R: GCCACCGTCGTCATCAGG |
|  | Purple acid phosphatase | *PAP*  [52] | TC152610 | XM_002464282 | 1745 | 0.0 | F: ATCCTTGTGGCGCTGTTCAC  R: CCTGAAGACCGACCAAGCTG |
|  | Thioredoxin | *Thioredoxin*  [53] | TC122013 | XM_002461523 | 791 | 0.0 | F: AGACAGAAATGGCGTCCGAG  R: AGTCAATGACCACCAGCTTGC |
|  | PEAMT | *PEAMT*  [54] | TC120366 | XM_002456149 | 1777 | 0.0 | F: TGTGGAATTGGTGGAGGTGAC  R: CCAATGGCACGCTCAAGTG |
|  | Calreticulin | *Calreticulin*  [55] | CF577421 | NM_001058334 | 475 | 1e-130 | F: CGAGTTCAGCAACAAGGACAAG  R: CATCAGACCCAAGCAATTTCAC |
|  | Putative stress-related protein | *Stress-related protein*  [7] | TC121856 | EZ052273 | 340 | 8e-90 | F: TAAAAGGTAGCCGCACGCAC  R: CGCAGCCGAACTTGAAGAAG |

*TC derived from Sugarcane Gene Index 3.0, remaining sequence IDs from Genbank (NCBI).

#Derived by BLASTn of non-redundant nucleotide database at NCBI on 20^th^ September 2010.
